# Sustaining student concentration: the effectiveness of micro-breaks in a classroom setting

**DOI:** 10.3389/fpsyg.2025.1589411

**Published:** 2025-08-06

**Authors:** Benjamin T. Sharpe, Michael Geoffrey Trotter, Beverley J. Hale

**Affiliations:** ^1^Institute of Psychology, Business and Human Sciences, University of Chichester, Chichester, United Kingdom; ^2^Department of Health and Sports, School of Health and Welfare, Halmstad University, Halmstad, Sweden; ^3^Institute of Sport, Nursing and Allied Health, University of Chichester, Chichester, United Kingdom

**Keywords:** vigilance, sustained attention, academic performance, cognitive load theory, spaced learning sustaining student concentration

## Abstract

This study investigates the impact of break frequency on students' attention and quiz performance during university classes, grounded in cognitive load theory and the concept of spaced learning. Involving 253 second-year undergraduates, it reveals significant effects of break conditions on performance, with micro-break participants outperforming others and sustaining better performance over time. The study employed a mixed-methods design, comparing traditional break periods with more frequent micro-breaks. Results showed that while performance declined across seminars for both conditions, aligning with vigilance literature, the micro-break condition exhibited more consistent performance. These findings contribute to our understanding of cognitive load management and the spacing effect in educational settings. The study highlights the importance of addressing attention spans in classrooms and suggests that incorporating micro-breaks may enhance students' engagement and academic achievement. Implications for instructional design in higher education are discussed, offering evidence-based strategies for educators to optimize the learning experience.

## Introduction

To address the issue of declining student attention in traditional classroom settings, it is essential to consider several factors. Disengaged students may perceive the traditional environmental setting as under-stimulating, and varying motivations for attending university may result in fluctuating levels of student engagement (Gijbels et al., 2005; Huxham, 2005; Lammers and Murphy, 2002; Miller et al., 2013). Moreover, passive learning approaches and classes that extend beyond the capacity of human cognition can contribute to reduced attention and engagement, regardless of class quality. Additionally, the pervasive and distracting nature of technology may capture students' attention and undermine their ability to focus (Ophir et al., 2009). Research has consistently demonstrated that human attention is limited to durations of up to 25 min (Risko et al., 2012; Sharpe et al., 2023, 2024, 2025; Thiffault and Bergeron, 2003; Verster and Roth, 2013; Young et al., 2009; Sharpe and Smith, 2024).

The importance of maintaining student attention in higher education can be understood through the lens of cognitive load theory (CLT; Sweller, 1988; Sweller et al., 2019). CLT posits that learning is most effective when the cognitive load on working memory is managed effectively. In a traditional classroom setting, extended periods of passive listening can lead to cognitive overload, potentially hindering the learning process. This aligns with Mayer's (2009) cognitive theory of multimedia learning, which emphasizes the limited capacity of working memory and the need for active processing to facilitate meaningful learning. Furthermore, the concept of spaced learning (Ebbinghaus, 1885; Bahrick and Hall, 2005) supports the potential benefits of micro-breaks. This theory suggests that information is better retained when it is studied in multiple, spaced-out sessions rather than in a single, prolonged session. Incorporating micro-breaks into classroom instruction may create natural spacing effects, potentially enhancing long-term retention of information.

Attention, for the current purposes, is defined as the ability to select effectively and allocate appropriate resources to process relevant information, whilst ignoring unnecessary stimuli (Petersen and Posner, 2012). In a university setting maintaining attention may be directed toward listening to the tutor, viewing the class slides, focusing on the current task, or engaging with class debate. Maintaining attention over prolonged periods, is termed vigilance (Davies and Parasuraman, 1982; Parasuraman and Davies, 1977). We acknowledge that “vigilance” is often used interchangeably with “engagement” in learning and teaching environments (Carini et al., 2006; Zepke and Leach, 2010). However, given no consistent unified definition of engagement (Axelson and Flick, 2010; Groccia, 2018), this paper adopted the term vigilance as meaning ability of individuals to maintain their focus of attention and to remain alert to stimuli over prolonged periods of time (Warm and Dember, 1998; Warm and Parasuraman, 1987).

It has been posited that individuals commonly experience attention lapses during prolonged periods (Killingsworth and Gilbert, 2010). A typical university class, which can range from 1 to 3 h depending on course scheduling and type (e.g., practical, theory), may hinder students' learning. Early research in laboratory settings has found that attention lapses occur in individuals in durations as little as < 30 min (Molley and Parasuraman, 2016; Teichner, 1974), 10 min (Temple et al., 2000), and even as early as the first 8 min of a task (Jerison and Pickett, 1963; Nuechterlein et al., 1983). More recent studies have reported declines in performance after the initial 25 min (Thiffault and Bergeron, 2003; Verster and Roth, 2013) and 10 min (Risko et al., 2012; Young et al., 2009). Such declines in performance, termed vigilance decrement, refer to “the decline in task performance resulting from sustaining attention over extended periods of time“ (Warm and Parasuraman, 1987, p. 623). Prolonged task performance can impair the retention, rehearsal, or recall of information, or hinder the ability to perform a skill or competency, potentially hindering later examination and practical performance.

Vigilance studies continue to investigate measures that potentially minimize vigilance decrement in tasks. For example, taking breaks, essentially resting between vigilance tasks (Lim et al., 2013; Ross et al., 2014); chewing gum during tasks (Morgan et al., 2014; Tucha and Simpson, 2011); engagement, between the observer and stimuli (Pop et al., 2012); and listening to music during tasks (Davies et al., 1973). Nevertheless, literature remains inconclusive on the attributes responsible for producing such decline in vigilance performance (Randall et al., 2014; Thomson et al., 2015). Early work discussing active learning and maintaining student concentration, based on the philosophy of constructivism, have suggested modes of teaching such as structured debates (Kumar, 2003), interaction with technology (Beekes, 2006), and small group discussions (Gibbs and Habeshaw, 1989) to name a few. Yet, with particular focus on teaching environments, the field of vigilance literature is surprisingly limited given the significance to education.

Young et al. (2009) found that students' concentration during a class declines similarly to that of a human operator monitoring automated equipment, with potentially negative consequences for learning and performance. To maintain attention and concentration, the authors recommend incorporating short breaks or novel activities, as previous literature suggests that changing task demands every 10–15 min may also help (Wankat and Oreovicz, 2003). Risko et al. (2012) demonstrated that, during a standard class, the ability to sustain attention decreases as a function of time. Further, the inability to sustain attention is negatively associated with memory for class material, and consequently reduces overall performance in laboratory reports, assignments, exams, and quizzes. As supported in previous vigilance literature (e.g., Ariga and Lleras, 2011), authors suggest that short rest breaks or task switches may show promise for maintaining student attention throughout a lecture or class (Bligh, 1998; Paulus et al., 2021; Smith, 2006). If these methods may in fact be effective in reducing the likelihood of a vigilance decrement, then such improvement may lead to improvements in the retention and understanding of material. Risko et al. (2012) suggest the method of assessing such technique could be in the form of media, interactive activities, and examinations. However, it is often noted that the time it takes for the onset of a vigilance decrement appears to be entirely dependent on the individual task characteristics and demands (See et al., 1995).

### The study aim

This study sought to investigate the influence of rest periods on class quiz performance. Comparisons were made to evaluate the degree to which a series of micro-breaks differ from a traditional break. We predicted a time effect on quiz performance, with performance decreasing over time. Such decline is consistently reported across previous literature (Risko et al., 2012; See et al., 1995; Thiffault and Bergeron, 2003; Verster and Roth, 2013). Following previous insight (Lim et al., 2013; Ross et al., 2014; Young et al., 2009), we predicted that more consistent quiz performance would result from the micro-break condition.

## Methods

### Participants

A total of 253 undergraduate students at a UK university took part in the study while taking a particular module. All students were English speaking first year second-semester students at the time of data collection. Data were collected across 2021 (*n* = 97) and 2022 (*n* = 156). The same tutor led the module for both cohorts. To ensure consistency across both academic years, identical module content, slide presentations, and timing protocols were maintained. The same tutor delivered all sessions using standardized presentation materials, and quiz structures remained identical across both cohorts. Ethical approval for the study protocol was awarded retrospectively by the lead institution. As such, demographic variables such as age and gender were not collected to maintain participant anonymity and to focus the analysis exclusively on cognitive capacity limitations rather than individual characteristics, consistent with our theoretical framework emphasizing universal attention span constraints.

### Study design, procedure, and instruction

The study lasted for 10 weeks and consisted of 10 seminar sessions that were held as part of a BSc year two psychology module. Each seminar session lasted 90 min with four parallel seminars each week. All parallel seminar sessions included 12 slides of content and were presented in the same order. The two conditions (micro-breaks and traditional) were carried out during these seminar sessions. The experimental design followed a systematic counterbalancing approach: each week, two of the four parallel seminar groups received micro-breaks while the other two received traditional breaks. This allocation pattern was rotated weekly to ensure all groups experienced both conditions equally across the 10-week period. As typical amongst UK universities, the “traditional” condition was a single 10-minute break 45 min into the seminar. Micro-breaks consisted of one 90-s micro-break every 10 min throughout the entirety of the seminar. To avoid any potential order effects, conditions were counterbalanced so that micro-breaks were integrated into two different seminar sessions per week (e.g., class 1 has micro-breaks in week 2, and regular breaks in week 3, etc.). This procedure was run for two consecutive cohorts. Participant engagement across all sessions was ensured through mandatory module attendance requirements and electronic quiz participation tracking, with completion rates exceeding 95% for all sessions. Content standardization was maintained through predetermined slide sequences and timing allocations, though we acknowledge that perceived difficulty may have varied across weeks as material progressed from foundational concepts to more complex applications, representing a potential confounding factor addressed in our limitations.

#### Condition instruction

During week 1, and as part of module content, all students were informed of the potential limitations of human attention and the supposed benefit of taking regular breaks during vigilance tasks through course material. Students were tasked to review the literature concerning sustained attention and vigilance decrements, and to collate methods that may facilitate student concentration. In addition to fulfilling the weeks learning objective, such material provided the introduction of micro-breaks to students and the subsequent purpose for doing so. The entire class was present for this overview. Students were then instructed what is meant by micro-breaks and provided the following instruction:

‘*Micro-breaks, within the current seminars, consist of doing anything other than directing attention toward the course material. This may involve closing your eyes, quietly speaking with fellow classmates, stretching, or drinking water, to name a few, over a period of precisely 90-seconds. A micro-break will commence at 10-minute intervals, as displayed on the slides, and conclude once the tutor begins speaking. You will be made aware at the beginning of all seminars whether micro-breaks are included in the seminar.'*

The inclusion of micro-breaks began in the second week of the module. Likewise, class quizzes began on the same week.

#### Performance task

During seminar sessions, class quizzes were administered in the final 10 min. Each quiz included one question per slide, presented in the original order of the material covered. For instance, if slide three covered Davies and Parasuraman's 1977 account of vigilance, then question three of the quiz pertained to that same topic. Participants were expected to choose the correct multiple-choice answer electronically using their own device through an online quiz tool (www.quizizz.com). To minimize discomfort, the end-of-class quiz scores were presented as a class average in consideration of the educational setting. After each selection, the tutor recorded the percentage of the class with the correct response. Each slide was covered for approximately 10 min, and percentage accuracy was utilized to represent performance at each timepoint of the seminar. The variable of time was referred to as “vigilance” for all analysis. The authors would like to clarify that the primary goal of the quiz for students was to serve as a session summary.

### Statistical analysis

Observed variables were screened for univariate normality using skewness and kurtosis ratios (Fallowfield et al., 2005; Kline, 1998). A two-way mixed design ANOVA was used to analyse the effect of condition (Micro-breaks vs. Control) and vigilance (performance across 12 questions per seminar) on percentage quiz performance. The alpha level (*p*) for statistical significance was set at 0.05 and partial eta squared (η*p*^2^) was used to measure effect size for all ANOVA analysis (Cohen, 1988). A Bonferroni adjustment was employed if multiple comparisons were justified to lower the significance threshold and avoid Type I errors (McLaughlin and Sainani, 2014). Violations of sphericity were corrected for by adjusting the degrees of freedom using the Greenhouse Geisser correction when epsilon was < 0.75 and the Huynh-Feldt correction when >0.75 (Girden, 1992). The statistical analysis was conducted using JASP software, version 0.12.2, an open-source analysis program freely accessible for use. All data is available upon request.

## Results

### Main effects

There was a significant main effect of condition on average quiz performance [F_(1, 78)_ = 254.698, *p*<*0.0*01, η*p*^2^ = 0.766]. The micro-break condition (M percentage = 65.13, SD = 13.26) showed better retention than the control condition (M percentage = 56.44, SD = 15.83; *p*<*0.0*01, *d* = 1.784). There was a significant main effect of vigilance on quiz performance [F_(8.270, 645.045)_ = 258.468, *p*<*0.0*01, η*p*^2^ = 0.768]. On average performance began to deteriorate as time progressed. Performance systematically deteriorated across time points, declining from 81.3% to 55.6% for the micro-break condition and from 82.5% to 51.5% for the traditional condition across the 12 time points within each session.

### Interaction effects

**Figure 1:**
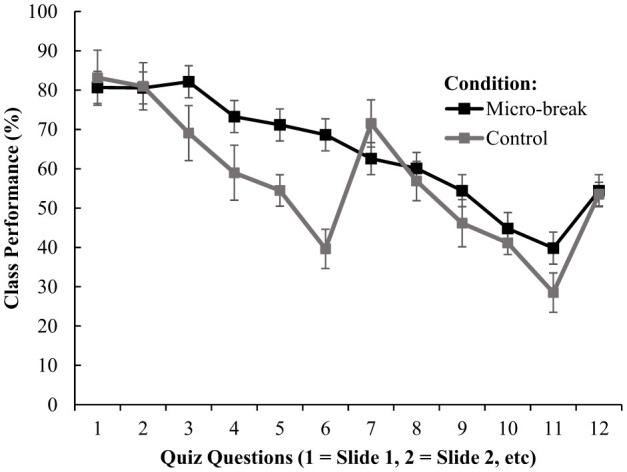
The influence of condition and time on quiz performance (with SE bars).

Vigilance had a 2-way significant interaction effect with condition [*F*_(8.270, 645.045)_ = 33.940, *p*<*0.0*01, η*p*^2^ = 0.303]. For the micro-break condition, significant declines in quiz performance from time point 1 began at time point 5 (*p*<*0.0*01) and continued to time point 12 (*p*<*0.0*01). The micro-break condition appeared to minimize the decline in performance between time points (e.g., time point 5 and 6 saw no significant decline in performance). For the control condition, significant declines in quiz performance from time point 1 began at time point 3 (*p*<*0.0*01) and continued to time point 12 (*p*<*0.0*01). A significant increase in performance was observed between time point 6 and 7 (given the traditional class break was provided between these points); however, the decline in performance continued immediately. Analysis of decline patterns revealed that both conditions experienced similar overall decline rates, with the micro-break condition declining at −3.73 percentage points per time point and the traditional condition at −3.52 percentage points per time point. However, the traditional condition demonstrated a substantial but temporary recovery effect at the mid-session break, with performance increasing from 39.6% at time point 6 (pre-break) to 71.2% at time point 7 (post-break), representing a 31.6 percentage point recovery. This benefit was immediately lost, with performance declining to 54.8% at time point 8, suggesting the traditional break provides only momentary respite rather than sustained improvement. In contrast, micro-breaks showed their greatest advantage during the critical middle period of sessions (time points 3–6), maintaining an average 20.6 percentage point advantage over the traditional condition, with individual time point advantages ranging from 14.7 to 30.1 percentage points. This pattern suggests that micro-breaks may be particularly effective at preventing the severe mid-session performance decline observed in the traditional condition, rather than simply providing temporary recovery. Irrespective of condition, declines in quiz performance were observed as time progressed (see Figure 1).

### Unplanned analysis

As material may have been delivered slightly differently between years, we briefly report the above analysis after separating the 2 years of study (see Table 1). The separate cohort analysis revealed consistent micro-break intervention effects across two distinct academic years, providing evidence for the robustness of the findings. Despite notable contextual differences between the cohorts, including the 2021 group representing the first post-COVID return to face-to-face learning with a smaller sample size (*n* = 97) vs. the 2022 cohort operating in a more normalized post-pandemic environment with greater statistical power (*n* = 156), both groups demonstrated similar patterns of results. When averaging across the entire module, there was no significant difference in overall performance percentage between the two cohorts (*p* > 0.5), suggesting comparable baseline academic abilities. In both years, micro-breaks produced substantial improvements in quiz performance compared to traditional break conditions, with effect sizes remaining consistently large across cohorts. The vigilance decrement phenomenon was reliably observed in both groups, with performance systematically declining over time regardless of the year, though the 2022 cohort showed an even more pronounced time effect. Most importantly, the interaction between break condition and time was significant in both cohorts, indicating that micro-breaks consistently provided differential protective benefits against performance decline throughout the seminar duration. The consistency of these interaction effects, which explained 30.7% and 32.2% of the variance in 2021 and 2022 respectively, suggests that the temporal advantage of micro-breaks over traditional breaks is a stable and replicable phenomenon that transcends specific contextual factors such as class size differences, post-pandemic learning disruptions, or year-specific variables. This robustness significantly strengthens confidence in the practical applicability and generalizability of micro-break interventions in typical university psychology seminar contexts, demonstrating that the cognitive benefits are not dependent on environmental circumstances but rather reflect fundamental attention and learning processes.

**Table 1:** Comparison of condition and vigilance on quiz performance.

**Cohort**	**Comparison**	**Test statistic (*F*)**	**Probability (*p*)**	**Partial Eta Squared (*ηp^2^*)**
2021	Condition	126.914	< 0.001	0.770
	Vigilance	125.411	< 0.001	0.767
	Condition × Vigilance	16.838	< 0.001	0.307
2022	Condition	117.768	< 0.001	0.721
	Vigilance	135.452	< 0.001	0.781
	Condition × Vigilance	18.044	< 0.001	0.322

## Discussion

The aim of this study was to examine the impact of two distinct types of rest periods on the academic performance of undergraduate students at the university level. Specifically, we sought to compare the effectiveness of traditional break periods vs. micro-breaks in enhancing overall class quiz performance question by question. The value of implementing regular rest periods has received considerable attention in the recent scholarly literature on student attention (e.g., Harris et al., 2021; Risko et al., 2012; Young et al., 2009). By analyzing the percentage of correct responses for each question across all classes, we aimed to investigate the influence of time on quiz performance. Our goal was to contribute to the existing literature on student vigilance and to assist educators in identifying a potential strategy for sustaining student attention by offering insights into the utilization of micro-breaks.

Our findings align with recent theoretical developments emphasizing that perfect sustained attention is fundamentally impossible due to inherent neural, biological, and cognitive limitations (Sharpe and Tyndall, 2025). Rather than attempting to overcome these constraints, our micro-break intervention demonstrates how educational practices can be designed to work harmoniously with the fundamental properties of human cognitive architecture. While there may have been variations in the delivery and reception of each weekly seminar (e.g., timing, tutor and student fatigue, practical material, debates), our data indicate a significant percentage difference in performance between the two conditions. Specifically, the data suggest a noteworthy improvement in quiz performance resulting from regular micro-breaks taken during seminar sessions. These findings align with previous research showing that rest breaks can enhance retention (Cepeda et al., 2009) and problem-solving abilities (see Sio and Ormerod, 2009 for a review), as well as increase energy and decrease subjective fatigue (Hunter and Wu, 2016; Zacher et al., 2014). Consistent with our hypothesis, the micro-break condition exhibited more consistent performance overall, although a decline in performance persisted throughout the end-of-class quiz for both conditions, with no significant differences apparent at the final question, where both conditions performed below 60 percent. The observed benefits likely reflect the optimization of attention management within cognitive constraints rather than the elimination of vigilance limitations, as neural mechanisms demonstrate that truly continuous attention is impossible due to rhythmic oscillations and the need for periodic default mode network activation (Fiebelkorn and Kastner, 2019; Raichle, 2015).

Beyond cognitive load reduction, the benefits of micro-breaks may involve multiple mechanisms including physiological arousal restoration, enhanced social engagement during brief interaction periods, and improved motivation through task segmentation. From a neurobiological perspective, micro-breaks may allow for the natural recovery of neurochemical systems, as both cholinergic and dopaminergic systems exhibit activity fluctuations that correlate with attentional performance, and GABAergic interneurons show adaptation effects requiring periodic recovery (Sarter et al., 2016; Cools and D'Esposito, 2011; Ferguson and Gao, 2018). While a decrease in executive processing resources is typically implicated in the vigilance decrement phenomenon (Head and Helton, 2015; Helton and Russell, 2015; Warm et al., 2008), our findings do not explain the overall reduction in quiz performance. Attentional theories suggest that task underload or the need for continuous stimulus processing can lead to cognitive resource depletion and declining student attention (MacLean et al., 2009; Matthews et al., 2017; Matthews and Davies, 1998). However, recent theoretical advances suggest that attention lapses are not failures to be eliminated but rather inevitable consequences of our adaptive cognitive design, reflecting the brain's need for periodic disengagement to maintain optimal functioning (Sharpe and Tyndall, 2025). Future research may wish to explore classroom vigilance regarding these attentional theories in the context of microbreaks (e.g., attentional resource theory; Grier et al., 2003; Helton et al., 2005).

Based on the data, it appears that there were significant declines in performance over time, which aligns with previous research on vigilance (Molley and Parasuraman, 2016; Risko et al., 2012; Swanson et al., 2012; Temple et al., 2000; Verster and Roth, 2013). These findings suggest that the ability to sustain attention is critical to vigilance performance. Sustained attention is a mechanism that facilitates the maintenance and engagement of a vigilance task (Robertson and Garavan, 2010). On average, a vigilance decrement was observed across all groups, regardless of condition, indicating a decline in this ability. This universal decline supports theoretical arguments that vigilance decrements reflect fundamental constraints of human cognitive architecture rather than individual deficiencies or inadequate training/effort (Sharpe and Tyndall, 2025). The data also suggest that the decline begins after the first 5 min and continues throughout the seminar, which is consistent with the observational work of Johnstone and Percival (1976). These findings strongly support the argument that educational design should acknowledge the theoretical impossibility of perfect sustained attention and focus on optimizing performance within these constraints rather than attempting to overcome them. The following findings may tentatively support the notion that tutors should present the most critical and core information during the initial portion of a class, with the aim of leveraging the class's initial capacity to retain information. The implementation of this strategy can potentially optimize the cognitive load of students, allowing them to better process and integrate new information with their prior knowledge. The previously discussed considerations may enable tutors to create a more engaging learning environment by capturing the students' attention and interest from the outset, which can facilitate active participation and critical thinking. Further research is necessary to establish the efficacy and generalizability of this approach across different subjects and student populations.

The implications of our findings extend beyond immediate classroom applications to broader questions about human-technology balance in educational settings. Recent research suggests that the optimal approach involves determining the appropriate balance between human capabilities and technological support, recognizing that humans excel in tasks requiring contextual understanding and pattern recognition while automated systems prove superior for maintaining vigilance during routine monitoring (Lundberg and Johansson, 2021; Parasuraman, 2020). The observed benefits of micro-breaks in this study can be interpreted through the lens of several pedagogical theories. From the perspective of cognitive load theory (Sweller et al., 2019), micro-breaks may serve to reduce extraneous cognitive load, allowing students to more effectively process and integrate new information. This aligns with Mayer's (2009) principles of multimedia learning, particularly the segmenting principle, which suggests that people learn better when a lesson is presented in user-paced segments rather than as a continuous unit. Moreover, the spacing effect (Bahrick and Hall, 2005) may explain why the micro-break condition showed more consistent performance. By introducing brief pauses throughout the lesson, students may have had opportunities for mini-review sessions, potentially reinforcing their understanding of the material. This is consistent with the testing effect (Roediger and Karpicke, 2006), which posits that the act of retrieving information strengthens memory more than additional study.

The findings of this study have significant implications for instructional design in higher education. They suggest that educators should consider restructuring their lessons to include regular, brief breaks, aligning with principles of active learning (Bonwell and Eison, 1991; Freeman et al., 2014). This approach may not only maintain student attention but also promote deeper processing and better retention of information. However, it is important to recognize that micro-breaks represent just one component of a broader approach to educational design that acknowledges cognitive limitations. As recent research emphasizes, the goal should not be to achieve perfect sustained attention but rather to create more sophisticated ways of working within our cognitive constraints (Sharpe and Tyndall, 2025).

While not as crucial as maintaining attention while operating a vehicle (e.g., Edkins and Pollock, 1997), a decrease in quiz performance could have significant consequences on students' overall grade if they are unable to recall essential concepts or material from class (Young et al., 2009). The recognition that attention limitations are fundamental rather than remediable has important implications for how we approach educational assessment and design, suggesting that traditional extended examination formats may themselves be misaligned with human cognitive capabilities. While it may be difficult to prevent a vigilance decrement during prolonged periods, research suggests that incorporating micro-breaks into the classroom setting could help mitigate this issue. This finding supports previous claims of a short attention span in the classroom, which have been noted in numerous pedagogical texts, and may help to explain why quiz scores were more consistent across sessions (e.g., Davies, 1993; McKeachie, 1986; Wankat, 2002). By providing students with more frequent opportunities to take short breaks, they may be able to maintain a more consistent level of attention, allowing them to actively engage with course material, view slides or practical demonstrations, and participate in discussions with their peers.

From an economic perspective, the implications of attention limitations provide compelling arguments for investing in appropriate educational support systems. Research in healthcare settings has shown that vigilance failures contribute significantly to errors, with associated costs exceeding billions annually, suggesting that continuing to rely primarily on human sustained attention is not only theoretically flawed but also financially unsound (Swanson et al., 2011; Reason, 2000). The decision to conduct an additional analysis by separating by cohort was prompted by the findings reported in literature indicating a significant influence of class size on student engagement, performance, and attitudes toward learning significantly (Gleason, 2012; Matta et al., 2015; Monks and Schmidt, 2011; Olson et al., 2011). Moreover, given that the 2021 cohort was immediately following the transition from remote to face-to-face teaching after the COVID-19 lockdown, it was reasonable to anticipate a potential impact on student behavior (see Ritchie et al., 2021; Ritchie and Sharpe, 2021 for discussion). However, our findings did not reveal any such differences, which suggests that student attention may be constrained by their cognitive capacity rather than the learning environment or contextual factors in the classroom. Therefore, instructional planning and delivery must account for such cognitive limitations.

### Limitations and future directions

Several important limitations must be acknowledged. First, potential confounding variables including individual differences in cognitive ability, motivation levels, and learning preferences were not controlled for and may have influenced results. Teaching style variations, even with the same instructor, could have affected engagement and performance across sessions. Additionally, the progressive increase in material complexity typical of academic modules may have contributed to the observed performance decline beyond vigilance effects alone. The generalizability of these findings beyond university psychology students requires careful consideration. Future research should examine micro-break effectiveness across different educational levels (secondary school, graduate programs), subject areas (STEM vs. humanities), and learning contexts (online vs. in-person instruction) to establish broader applicability.

As demonstrated repeatedly in literature, beyond educational settings, rest breaks are generally assumed to decrease vigilance decrement and, in some contexts, replenish cognitive resources and subsequent performance (Ross et al., 2014). As such, it may not be surprising that our study observed a benefit from micro-breaks. The incorporation of such strategy, however, must not be considered a sole means maintain student attention. Tutors should continue to utilize numerous active learning pedagogical strategies that follow constructivist perspectives of learning (e.g., Carr et al., 2015; Chi and Wylie, 2014) and seek to promote more active and engaged learning (see Bonwell and Sutherland, 1996). Likewise, future research may explore different types of micro-breaks beyond our unstructured and seated variation. Previous work has demonstrated some significant benefits of physical activity on fatigue (Meier and Welch, 2016; Watling et al., 2014), while relaxation techniques have recently been found to hold numerous benefits that may support academic performance (Blasche et al., 2018; Sianoja et al., 2018). Future investigations should incorporate direct measures of cognitive load, attention, and physiological arousal to provide stronger mechanistic validation of our findings. Individual difference factors such as working memory capacity, baseline attention abilities, and motivation should also be systematically examined to identify students who may benefit most from micro-break interventions.

The authors recognize that by focussing on group quiz performance we reduced the richness of data (i.e., individual differences in quiz performance) and excluded possible insight into those that may excel irrespective of the conditions; for example, if a sub-group of the sample consistently outperformed their classmates. In fact, previous literature has suggested numerous cognitive abilities appear to facilitate sustained performance in vigilance tasks (Furley and Memmert, 2012; Unsworth et al., 2012), this would be worthy of future exploration. Likewise, any variation in performance throughout a quiz may be reflective of the cognitive state during a particular part of the seminar session. With respect to the mind-wandering, which typically refers to the failure to hold attention on a primary task and instead attention shifts toward task-unrelated thought (McVay and Kane, 2009; Smallwood and Schooler, 2006), perhaps students' attention is consistently fluctuating toward the primary goal and toward internal thought (see Risko et al., 2012 for detailed account). Not only has mind-wandering been demonstrated to impairment comprehension during reading tasks (Dixon and Bortolussi, 2013; Jackson and Balota, 2012; McVay and Kane, 2012a) and distraction during lectures (Farley et al., 2013; Szpunar et al., 2013), but the frequency of such lapses of attention been correlated with the quality of note taking, course interest, and performance in course examinations (Lindquist and McLean, 2011). Of interest, and in line with the executive failure hypothesis, literature reports individuals with greater cognitive ability (e.g., working memory capacity) show a lower tendency to mind-wander (McVay and Kane, 2009, 2012a,b). Future research may wish to replicate the current study design and explore such individual variability in cognitive ability amongst students.

## Conclusion

The study sought to investigate the influence of two types of break periods on class quiz performance. Findings support earlier documentation that a vigilance decrement will be observed across tasks of prolonged duration while showing that the inclusion of more regular but shorter break periods offsets some of the negative consequences of traditional class lengths. These findings contribute to literature on improvement of student concentration and reduction of learning fatigue. The current findings present a possible alternative to aid student attention and provide several suggestions for further investigation. While our results demonstrate clear benefits of micro-breaks in university psychology seminars, broader implementation should consider contextual factors, individual differences, and the integration of multiple evidence-based teaching strategies. This study contributes to the growing body of literature on evidence-based teaching practices in higher education (Ambrose et al., 2010). By demonstrating the potential benefits of micro-breaks, it provides educators with a practical, theory-grounded strategy for enhancing student learning. Future research could explore how this approach interacts with other evidence-based teaching methods, such as retrieval practice (Karpicke and Blunt, 2011) or collaborative learning (Johnson et al., 2014), to further optimize the learning experience in higher education settings.

## Data Availability

The raw data supporting the conclusions of this article will be made available by the authors, upon reasonable request.
